# Hydrostatic and osmotic pressure study of the RNA hydration

**DOI:** 10.1007/s11033-012-1452-z

**Published:** 2012-02-08

**Authors:** Małgorzata Giel-Pietraszuk, Jan Barciszewski

**Affiliations:** Institute of Bioorganic Chemistry, Polish Academy of Sciences, Noskowskiego 12/14, 61-704 Poznań, Poland

**Keywords:** Leadzyme, tRNA^Phe^, High hydrostatic pressure, RNA hydration, RNA structure

## Abstract

The tertiary structure of nucleic acids results from an equilibrium between electrostatic interactions of phosphates, stacking interactions of bases, hydrogen bonds between polar atoms and water molecules. Water interactions with ribonucleic acid play a key role in its structure formation, stabilization and dynamics. We used high hydrostatic pressure and osmotic pressure to analyze changes in RNA hydration. We analyzed the lead catalyzed hydrolysis of tRNA^Phe^ from *S. cerevisiae* as well as hydrolytic activity of leadzyme. Pb(II) induced hydrolysis of the single phosphodiester bond in tRNA^Phe^ is accompanied by release of 98 water molecules, while other molecule, leadzyme releases 86.

## Introduction

**Fig. 1:**
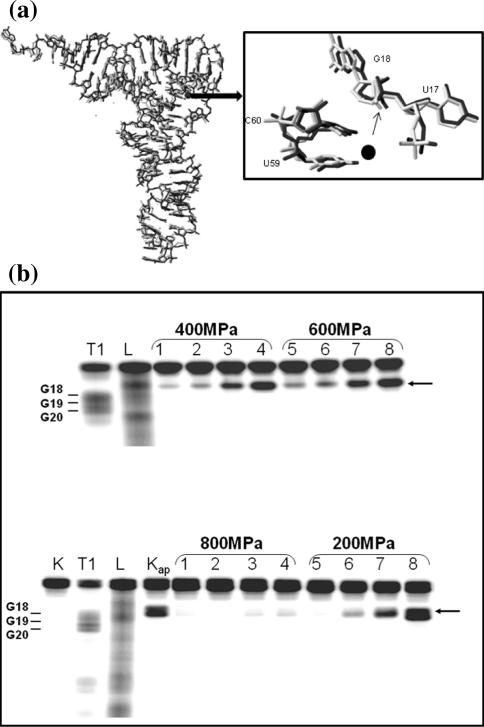
**a** Superposition of yeast tRNA^Phe^ structure—*grey* (PDB: 1TRA) and tRNA^Phe^ with Pb^2+^—*black* (PDB: 1TN2), in separate *box* is shown the details of reaction site structure; **b** HHP effect on Pb^2+^ induced yeast tRNA^Phe^ hydrolysis. The autoradiogram of the 10% PAGE with 7 M urea of the cleavage reaction of 6.7 μM (300,000 cpm) [3′-^32^P]tRNA^Phe^ induced with 0.5 mM Pb^2+^ in 10 mM Tris–HCl pH 6.8, 10 mM MgCl_2_ and 0.4 M NaCl at 22°C, *L* alkaline ladder; *T1* limited hydrolysis with RNase T1; *K* control without Pb^2+^; *K*_*ap*_ hydrolysis with Pb^2+^ at AP for 120 min; at 400, 600, 800 and 200 MPa, *1*–*4* respectively at 1, 2, 4 and 16 h. *Arrows* depict location of the cleavage product on the gel, *ap* ambient pressure (0.1 MPa)

The three-dimensional structure of nucleic acids results from an equilibrium between electrostatic interactions of negatively charged phosphates, stacking interactions of nucleic acid bases, hydrogen bonds between polar atoms and water surrounding the molecule, as well as the conformational energy of the sugar–phosphate backbone [1]. A key role in the structure of nucleic acids plays water [2]. Dehydration of DNA results in a conformational change from B-form to A-form [3]. Although hydration and its role in macromolecular structure and activity has been extensively studied in the case of RNA still many questions remain unanswered [4]. To shed more light on that problem, we examined hydration changes which accompany hydrolysis of yeast phenylalanine specific transfer RNA (tRNA^Phe^) with Pb(II). Yeast tRNA^Phe^ can be very useful model for this study because its three-dimensional structure has been determined in many crystallographic studies (Fig. 1a) [5–8]. Moreover it undergoes specific lead(II) induced hydrolysis between D17 and G18 [9]. To solve the problem we analyzed the hydrolysis reaction at high hydrostatic and osmotic pressure (OP).

High hydrostatic pressure (HHP) is a convenient tool for study hydration because it allows to induce changes without addition external agents, e.g., salts. Both HHP as well as OP affect the structure of water [10, 11].

The mechanism of that cleavage proposed on the basis of crystal structure of yeast tRNA^Phe^ soaked with Pb(II) starts with abstraction of H^+^ from a ribose 2′-OH group by Pb(II) bound hydroxyl group. RNA hydrolysis is pH dependent and the optimum for Pb(II) is 7.5 while for Mg(II) is 8.5 [12–14]. The Pb(II) ion bound in the D-loop replaces a Mg(II) from its site located in close vicinity to it in the native structure [15]. The molecule of tRNA is extensively hydrated. In the crystal structure of yeast tRNA^Phe^ 120 water molecules were precisely identified that interact directly with the molecule, about 14% bind adjacent or non-adjacent phosphate atoms, 10% 2′-OH of the ribose and either the N3 of purines or the O2 of pyrimidines [5]. Molecular dynamics analysis showed ca. 820 water molecules in first hydration layer of tRNA, while 269 were estimated for h = 0.2 g—the value determining boundary below which the conformational motion are strongly suppressed (h—hydration in grams of water per g of tRNA) [16]. Most of water molecules in tRNA are clustered around Mg^2+^ ions and regions of strong negative electrostatic potential near the junctions between D and T loops [16]. X-ray analysis of the 7 bp long tRNA^Ser^ acceptor-stem structure showed that hydration shell consists of 75 water loci per duplex [17]. Many of the interactions observed between EF-Tu-GTP and aatRNA backbone clearly involve the solvent as integral part [18].

Other RNA that specifically binds Pb(II) and exhibits hydrolytic activity was found by in vitro selection method [19, 20]. Its consensus sequence motif called the “leadzyme” consists of a short duplex with an internal bulge loop. Leadzyme activity depends on Pb^2+^ only [21–23]. Leadzyme forms an A form duplex with one non-Watson–Crick C–A base pair flanking the 5′-end of three-nucleotide bulge, 5′-GAG-3′ at substrate strand. The scissile phosphodiester bond is located at the junction between helical part and the bulge. The first step of RNA hydrolysis catalyzed with leadzyme proceeds according to mechanism proposed for tRNA^Phe^, yielding a free 5′-hydroxyl and a 2′,3′-cyclic phosphodiester. That step is analogous to the products of reaction catalyzed by natural small ribozymes such as the hammerhead and hairpin, but in the second step the cyclic phosphate is hydrolyzed to a 3′-phosphate in a manner similar to ribonuclease A. This is not observed in neither for tRNA^Phe^ nor the hammerhead or hairpin ribozyme reaction [24–26].

In the paper we show that HHP inhibits the hydrolysis of tRNA^Phe^ with Pb(II), but does not affects significantly a spontaneous cleavage (not Pb(II) dependent) of double stranded RNA. In contrast to hammerhead and HDV ribozymes, leadzyme was not active without Pb^2+^ at HHP. Hydrolysis of single phosphodiester–sugar bond of tRNA^Phe^ and leadzyme targeted RNA is accompanied by release of 98 and 86 water molecules as estimated by osmotic stress, respectively.

## Materials and methods

### RNA synthesis

Reaction was performed on a PCR-Mate EP model 391 DNA synthesizer (Applied Biosystems) with 2-0-triisopropylsilyl-protected phosphoramidite synthons. After deprotection, the oligoribonucleotides were desalted on a Qiagen Tip 500 column as described earlier [27]. This method was used for the preparation of the following oligonucleotides: L1: 5′-GGACGAGCCAG; L2: 5′-CUGGGAAGUCC.

### 3′-end RNA labeling

6 μL of mixture containing 0.16 mM Cp, 20 μCi [γ-^32^P]ATP and 10 U T4 polynucleotide kinase and 1 × PNK buffer (Promega) was incubated at 37°C for 45 min and 99°C for 2 min. Than 0.8 nmol of tRNA^Phe^ and 10 U of T4 RNA ligase (Boehringer) in of 20 μL of ligase buffer (50 mM Tris-HCl pH 8.2, 5 mM ATP, 10% DMSO, 20 μg/mL BSA, 30 mM DTT, 10MgCl_2_,) were added and incubated 16 h at 4°C.

### 5′-end RNA labeling

5 μg of RNA was mix with [γ-^32^P]ATP and 6 U T4 polynucleotide kinase (Promega) and kinase buffer (Promega) and incubated 30 min at 37°C. Reaction mixture was then separated in a 15% (tRNA) and 24% (L1) polyacrylamide gel with 7 M urea. The radioactive band was cut out, the RNA was eluted with buffer and precipitated with ethanol [28].

### Lead induced hydrolysis of tRNA^Phe^

A mixture (49.6 μL) composed of 3′-end-labeled tRNA^Phe^ (300,000 cpm), tRNA^Phe^ (30 μg), 27.5 μL NaCl (0.1 M) and 7 μL Tris-HCI pH 6.8 (0.1 M) was incubated for 2 min at 70°C. Then, 7 μL of MgC1_2_ (0.1 M) was added, and samples were cooled slowly (15 min) to 22°C after that 15.4 μL of Pb(CH_3_OO)_2_ was added to final concentration of 0.5 mM.

### The leadzyme catalyzed hydrolysis of RNA

Reaction was carried out in 15 mM MOPS (3-(*N*-morpholino)propanesulfonic acid) pH 7.5 at 22°C. The labeled L1 (30000 cpm, 1 mM) and L2 (1 mM) were mixed together and heated up to 60°C for 2 min, cooled slowly (1°C/min) and incubated at 22°C for 30 min. The cleavage reaction was initiated by the addition of Pb^2+^ (25 mM) and carried out at 22°C. Reaction was stopped by addition of an equal volume of loading buffer (25 mM sodium citrate pH 5, 1 mM EDTA, 7 M urea, 0.1% xylene cyanol, and 0.1% bromophenol blue) and stored on ice.

### Analysis of HHP effect on hydrolysis reaction

The effect of HHP on Pb(II) induced hydrolysis of tRNA^Phe^ was investigated by subjecting 15 μL of the reaction mixtures to pressure ranging from 0.1 to 800 MPa. Aliquots were removed after 1, 2, 4, 16 h and quenched with loading buffer and analyzed on 10% PAGE.

For analysis of the influence of HHP on leadzyme catalyzed hydrolysis of L1 15 μL of reaction mixture was subjected to pressure ranging from 0.1 to 800 MPa. Aliquots were removed, and reaction was quenched at various times (0–90 min at 0.1 MPa and 60–180 min at 200–800 MPa).

For technical reasons the time necessary to load the chamber, achieve and release a desired pressure after experiment and stop the reaction takes 10 min. The control reaction was carried out in time necessary to operate the HHP apparatus to subtract the hydrolysis carried out during that time. Every analysis was repeated three times. The radioactivity level present in samples was measured and approximately the same value was loaded on the gels.

Pressuring was carried out in Teflon vessels placed in a high pressure cell (Unipress, Warsaw).

### Kinetics of the cleavage reaction at hydrostatic pressure

The activation volume of the reaction (ΔV^≠^) was calculated from the equation: k = A exp −(PΔV^≠^/RT). Using the software Kleidagraph, the kinetics toward equilibrium were fitted to the exponential equation: x = x_eq_(1−e^−kobs t^), where x_eq_ is the fraction of cleaved RNA at equilibrium. The reaction volume change (ΔV) was calculated using the equation: K = A exp −(PΔV/RT), k and K are the rate and the equilibrium constants of the reaction respectively, R is the universal gas constant (8.314 cm^3^ MPa °K^−1^ mol^−1^), T the temperature (°K) and P the pressure (MPa) [29].

### Kinetics of the cleavage reaction under OP

The effect of OP was analyzed by including OP agents, namely polyethylene glycol 400 in the cleavage medium, to final concentrations 0, 2.5, 5, 7.5% (v/v). Aliquots were removed at different time (30–120 min), quenched with loading buffer and analyzed on 24% for L1 and 10% PAGE for tRNA^Phe^. The number of water molecules released upon RNA cleavage was calculated using the equation: δ*k*Tln(k^Π^/k^0^)/δΠ_osm_ = ΔV_w_ = ΔN_w_ (30 Å^3^); k^Π^ is the observed cleavage rate constant (k_obs_) at OP Π, k^0^ is the k_obs_ in the absence of added solute. *k* is the Boltzmann constant and T the temperature (°K). ΔV_w_ is the change of water volume (30 Å^3^ the molecular volume of water and ΔN_w_ change in the number of associated water molecules) [29].

### Electrophoresis, autoradiography and calculations of hydrolysis yield

tRNA^Phe^ and L1 hydrolysis products were analyzed on 10 and 24% polyacrylamide gels with 7 M urea, pH 8.3, respectively. The limited hydrolysis of tRNA^Phe^ and L1 with T1 RNase under denaturing conditions was done in 20 mM sodium acetate buffer pH 4.5, containing 7 M urea and 1 mM EDTA for 20 min at 55°C [28]. Alkaline hydrolysis was carried out in 50 mM NaOH and 1 mM EDTA for 1 min in case of tRNA^Phe^ and 18 min for L1. For quantitative analysis, the radioactivity of ^32^P on the gels was counted in a Typhoon 8,600 Imager with ImageQuant software (Molecular Dynamics). The rate constants were calculated by plotting the natural log of the amount of cleaved substrates versus time (5–120 min).

## Results and discussion

HHP reversibly modifies hydrophobic and ionic interactions and finally changes the solvation of macromolecules. It can modify the equilibrium constant of a reaction (K_eq_) if it is accompanied by a significant volume change (ΔV). It can also influence the velocity of reactional processes that involve a significant activation volume (ΔV^≠^). These parameters (ΔV and ΔV^≠^) can be directly measured from variation of the reaction equilibrium and rate constants as a function of pressure [30, 31]. ΔV and ΔV^≠^ values allow to discuss changes in the hydration of the molecule effected by conformational changes caused by single bond breakage [31]. OP can also affect the hydration of molecule which results in conformational changes of the enzyme and thus its activity. Analysis of these changes provides information about changes in hydration [29].

**Fig. 2:**
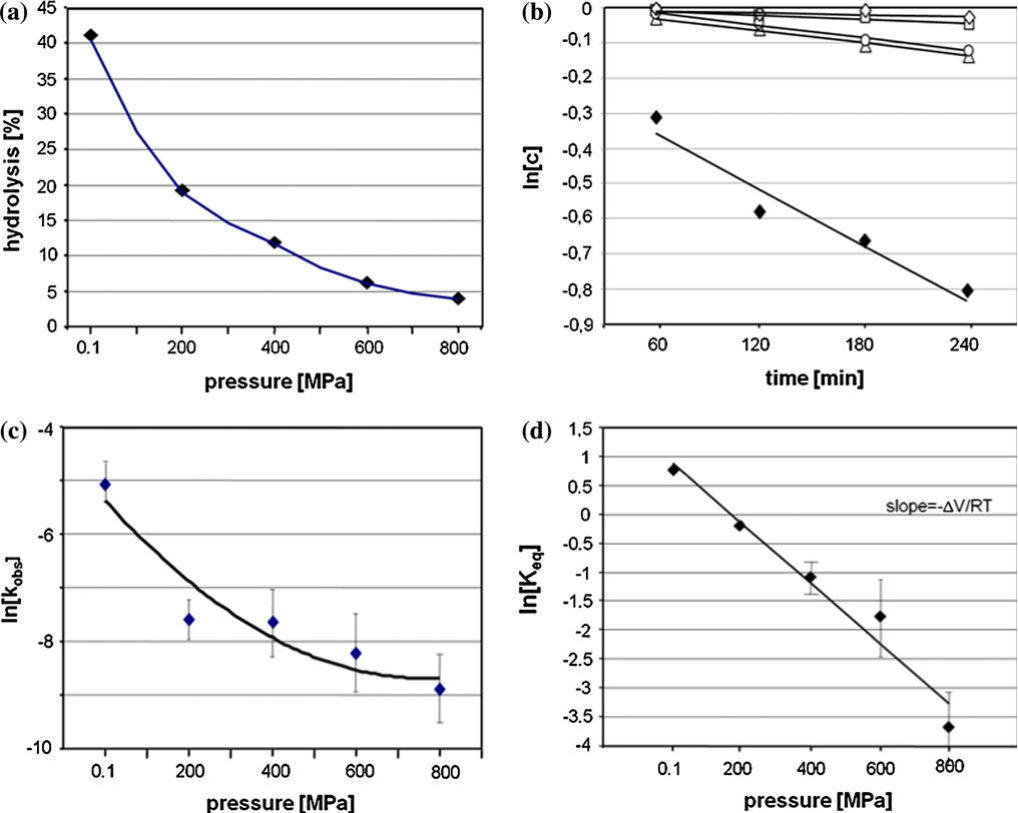
HHP dependence of yeast tRNA^Phe^ cleavage induced with Pb^2+^. **a** Hydrolysis of tRNA^Phe^ with Pb(II) dependent on pressure carried out for 2 h. **b** Determination of k_obs_ by plotting the natural logarithm of product concentration at pressure: 0.1 MPa (*filled* *diamonds*). 200 MPa (*open triangle*), 400 MPa (*open circle*), 600 MPa (*open square*), 800 MPa (*open diamonds*) as a function of time. **c** Determination of the activation volume changes associated to the hydrolysis reaction. The ΔV^≠^ was calculated from the plot by curve fitting to equation: lnk_obs_ = lnk_0_ − ΔV^≠^P/RT + Δβ^≠^P^2^/2RT. **d** Linear decrease of the theoretical equilibrium constants logarithms with increasing hydrostatic pressures. K_eq_ was calculated as the ratio of cleaved and uncleaved fractions at equilibrium. The percentages of cleaved products were obtained from the extrapolation of each exponential. Change of the reaction volume (ΔV) was calculated from the slope of the curve RTlnK_eq_/pressure

In our study we used yeast tRNA^Phe^ which is specifically cleaved with Pb(II) ions between D17-G18 (major cleavage) however some minor cleavages appear as well at D16, D17, G19 and G20 depending on reaction condition [12–15]. Our experiments were carried out in the way which allows obtaining mainly a single cleavage at HHP, although the cleavage at G19 was still observed at 200 MPa. Lead(II) induced tRNA^Phe^ hydrolysis reaction carried out at ambient pressure (AP) for 120 min proceeds with 43% yield (Figs. 1, 2a) with the observed cleavage rate constant (k_obs_) of 6.3 × 10^−3^ min^−1^ (Table 1; Fig. 2b). To determine the effect of HHP on tRNA^Phe^ cleavage with Pb^2+^ the reaction mixture was pressured from 0.1 to 800 MPa for 60–240 min (Fig. 2a, b). HHP decreases considerably the k_obs_ (Table 1) of Pb(II) induced hydrolysis which is reflected by positive value of ΔV^≠^. In the case of the non-linear plot of lnk_obs_ versus pressure (Fig. 2c) k_obs_ can be expressed by the quadratic equation [32]:

$$ {\text{InK}}_{\text{obs}} = {\text{InK}}_{ 0} - \frac{{\Updelta {\text{V}}^{ \ne } }}{\text{RT}}{\text{P + }}\frac{{\Updelta \beta^{ \ne } }}{{ 2 {\text{RT}}}}{\text{P}}^{2} $$

where Δβ^≠^ is the activation isothermal compressibility. To understand the relevance of compressibility. One should remember that the partial molar volume of a macromolecule in solution is the sum of: the constitutive atomic volumes (V_a_), the volume of cavities in the interior due to imperfect atomic packing (V_c_) and the volume change of the solvent due to hydration of solvent-accessible amino-acid groups (ΔV_h_) [32]. Activation volume, calculated from the plot by curve fitting to above equation, amounts 37.6 cm^3^/mol while Δβ_app_^≠^ 0.064 cm^3^/mol. The extrapolation of kinetic data allowed to estimate the equilibrium constant (K_eq_) at each pressure (Fig. 2d) and calculate ΔV, which amounts 10.2 cm^3^/mol.

**Table 1:** Kinetic and volumetric parameters for RNA hydrolysis at pressure 0.1–800 MPa

p (MPa)	k_obs_ × 10^−3^ (min^−1^)		
	tRNA^Phe^	L1 specific hydrol.	L1 spontaneous hydrol.
0.1	6.30	10.0	0.84
100	–	0.72	0.30
200	0.50	0.18	0.20
300	–	0.12	0.16
400	0.48	0.08	0.13
600	0.26	–	0.08
800	0.13	–	0.03
ΔV^≠^/ΔV (cm^3^/mol)	39.5/10.2	30.0/24.9	10.5/2.5
ΔV_w_ (cm^3^/mol)	−1,760	−2,100	−540
Water released	98	86	30

**Fig. 3:**
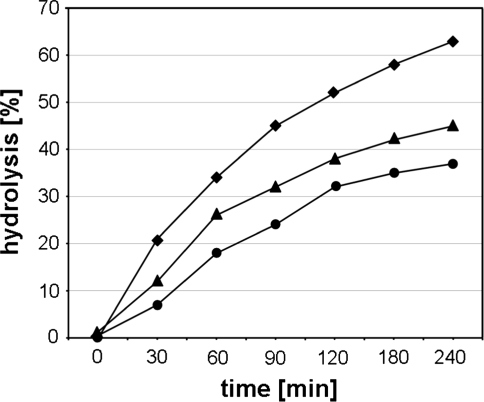
Reversibility of the effects of HHP on yeast tRNA^Phe^ hydrolysis with Pb(II). Kinetics of reactions carried out at AP (*diamonds*) and after 2 h of reaction under pressure 200 MPa (*triangle*) and 800 MPa (*circles*). The reaction mixtures were exposed to AP and allowed to react for 4 h

Analysis of the tRNA^Phe^ hydrolysis at 0.1 MPa followed the pressuring at 200 and 800 MPa for 2 h showed that the reaction is almost fully reversible (k_obs_ amounts 6.2, 6.0 and 5.8 × 10^−3^ min^−1^ for 0.1, 200 and 800 MPa, respectively) (Fig. 3). It means that observed decrease of hydrolysis at HHP does not result from irreversible changes in tRNA structure.

**Fig. 4:**
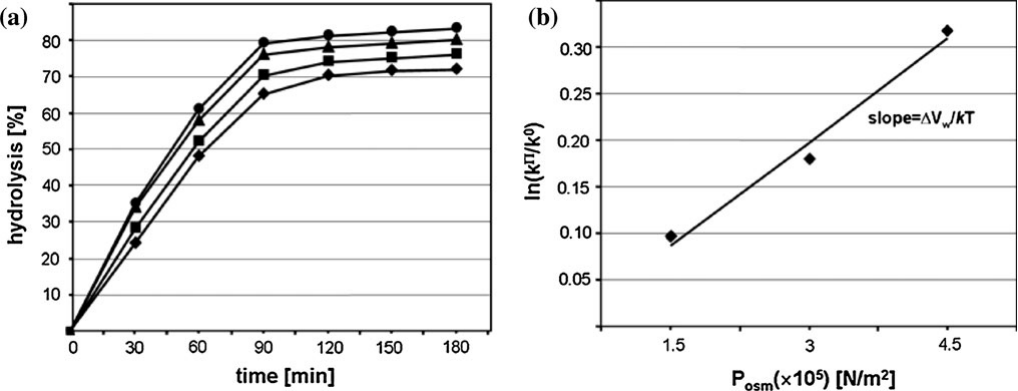
OP dependence of tRNA^Phe^ hydrolysis catalyzed with Pb(II). **a** Hydrolysis of tRNA^Phe^ in the presence of increasing concentrations of PEG 400: 0% (*diamonds*), 2.5% (v/v) (*squares*), 5% (*triangles*), 7.5% (*circles*); **b** effect of OP on the observed cleavage rate constant: ln(k^Π^/k^0^) plotted as a function of OP. k^Π^ and k^0^, the observed rate constant of the reaction under osmotic stress and in standard conditions, respectively; *k* the Boltzmann constant, *T* the absolute temperature (K)

Analysis of reaction kinetics carried out in condition of osmotic stress enables to calculate the number of exchanged water molecules [29]. OP acts opposite to HHP which is reflected by negative value of ΔV_w_ = −1,760 cm^3^/mol (Fig. 4a, b) corresponding to 98 water molecules released from tRNA^Phe^ molecule as a consequence of Pb(II) induced cleavage. After cleavage, tRNA still migrates as a single band on non-denaturing gel (data not shown) which means that the structure is fully stabilized by hydrogen bonds. Analysis of free tRNA^Phe^ crystals soaked with Pb(II) has shown that ion binding does not induce significant structural changes, however superposition of that molecule with free tRNA^Phe^ shows small differences in atom position (Fig. 1a) but in the crystal the cleavage of the P–O bond was not detected [13]. FTIR spectra of yeast tRNA^Phe^ recorded up to 1,400 MPa showed an increase of molecule hydration and some conformational changes [33]. Similarly analysis of DNA at pressure up to 1,380 MPa showed only subtle changes in its structure and increase of the number of bound water molecules from 57 to 76 [34]. However, change in the number of water molecules associated with tRNA^Phe^ must be accompanied by change in the nucleic acid conformation.

It was shown that the Pb(II) induced cleavage between D17 and G18 occurs in tRNA consisting of two (not ligated) fragments 1–36 and 38–76. The prerequisite for hydrolysis is, however, the presence almost whole molecule having both TΨC and DHU [35]. It means that this reaction depends on proper three-dimensional structure of tRNA^Phe^.

To evaluate whether the observed amount of water expelled from tRNA molecule was located rather in the vicinity of the reaction site or scattered throughout the whole body of tRNA we used smaller RNA molecule. Because in the case of yeast tRNA^Phe^ specific cleavage with Pb(II) is highly structure dependent and occurs only in the presence of whole molecule, any fragment of tRNA could not be used.

**Fig. 5:**
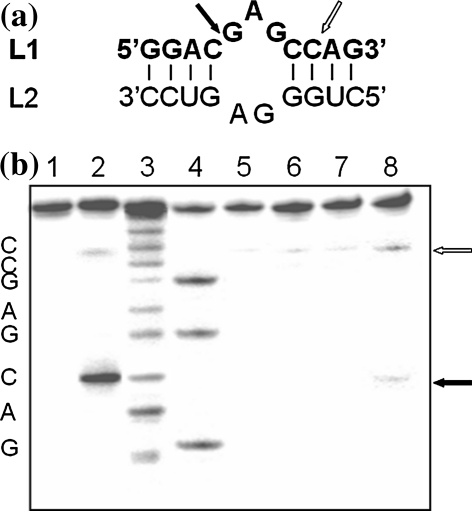
Secondary structure of leadzyme construct used in the work. **a** *Black arrow* points the position of leadzyme catalyzed hydrolysis, *open arrow* shows spontaneous cleavage. **b** 24% PAGE with 7 M urea L1 substrate hydrolysis catalyzed with L2 at HHP. The hydrolysis reaction of 1 mM [^32^P] labeled L1 with 1 mM L2 was carried in 15 mM NaMOPS pH 7.5 buffer containing 25 mM Pb^2+^. Lanes: *1* control, *2* reaction at 0.1 MPa for 2 h, *3* alkaline hydrolysis, *4* limited hydrolysis with RNase T1, *5*−*8* hydrolysis of L1 at 400 MPa for 0.5, 1, 2 and 4 h

**Fig. 6:**
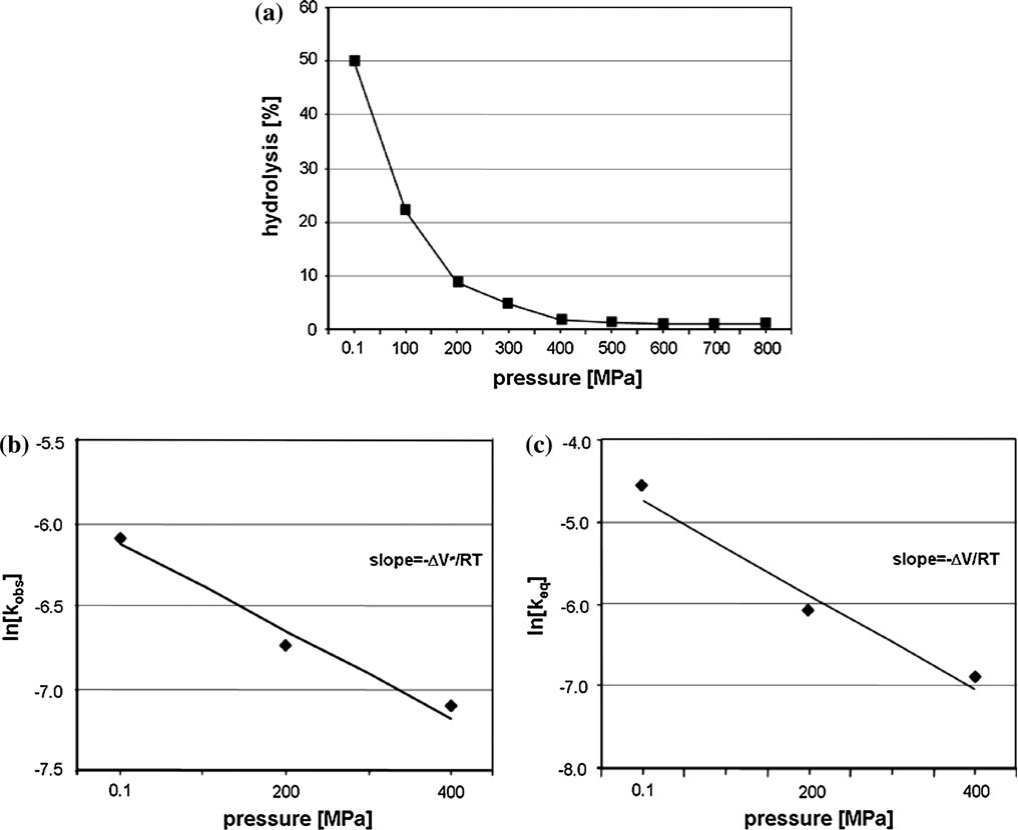
Hydrostatic pressure dependence of the leadzyme catalyzed cleavage reaction of L1. **a** Carried out in MOPS containing Pb(II) for 2 h. Determination of the activation volume (ΔV^≠^) (**b**) and volume change (ΔV) **(c)** accompanying the specific hydrolysis reaction, calculated from the slope of the curve

The only known molecule, except of yeast tRNA^Phe^, which is specifically hydrolyzed with Pb(II) is leadzyme. The one used in our work consists of an asymmetric internal loop of five-purine nucleotides surrounded by two short double RNA helices (Fig. 5a). It catalyzes specific hydrolysis of its target L1 at a C-G phosphodiester bond on the longer fragment of the loop (Fig. 5a, b) [19, 20, 27]. The yield of that cleavage at 0.1 MPa reaches a maximum of 52% after 2 h with k_obs_ of 1 × 10^−2^ min^−1^ (Table 1). An increase of hydrostatic pressure up to 800 MPa decreases the hydrolysis efficiency (Fig. 6a; Table 1). In contrast to tRNA^Phe^ leadzyme catalyzed reaction is almost completely inhibited at 400 MPa therefore calculation was carried out in the range 0.1–400 MPa. In that pressure range the plots of lnk_obs_ and lnK_eq_ versus pressure are linear. The ΔV^≠^ and ΔV amount 30.0 and 24.9 cm^3^/mol, respectively (Fig. 6b, c; Table 1).

**Fig. 7:**
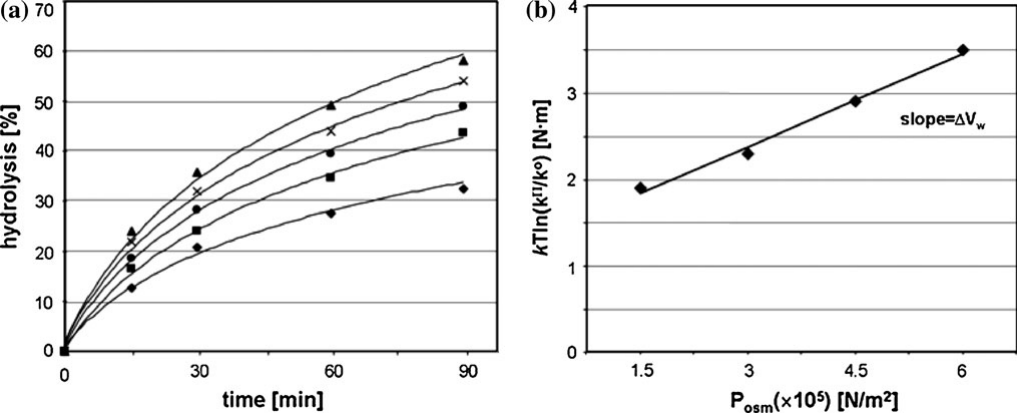
OP dependence of leadzyme catalyzed hydrolysis reaction of L1. **a** Kinetics of cleavage reaction was carried out in increasing concentration of PEG 400: 0% (*diamonds*), 2.5% (v/v) (*squares*), 5% (*circles*), 7.5% (*crosses*) and 10% (*triangles*). **b** Determination of ΔV_w_ for Pb(II) catalyzed L1 hydrolysis

**Fig. 8:**
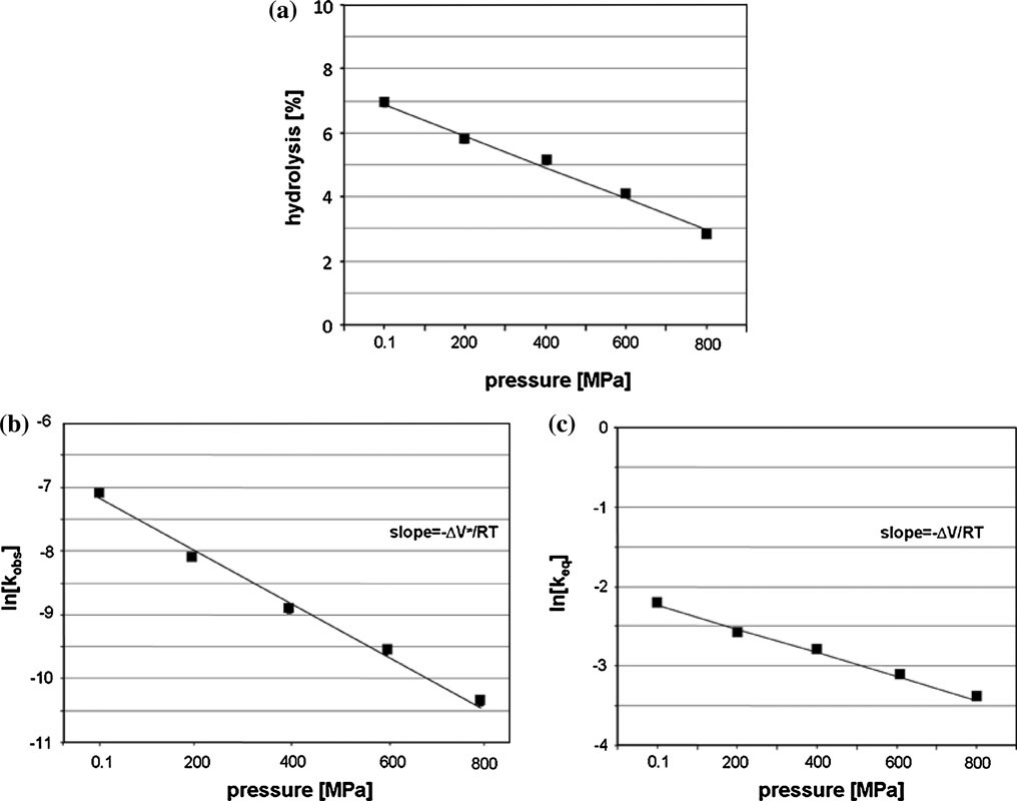
Hydrostatic pressure dependence of spontaneous hydrolysis of L1 (**a**) carried out in MOPS without Pb(II). Determination of ΔV^≠^ (**b**) and ΔV (**c**) from the slope of ln[k_obs_] and ln[K_eq_] plotted as a function of pressure

**Fig. 9:**
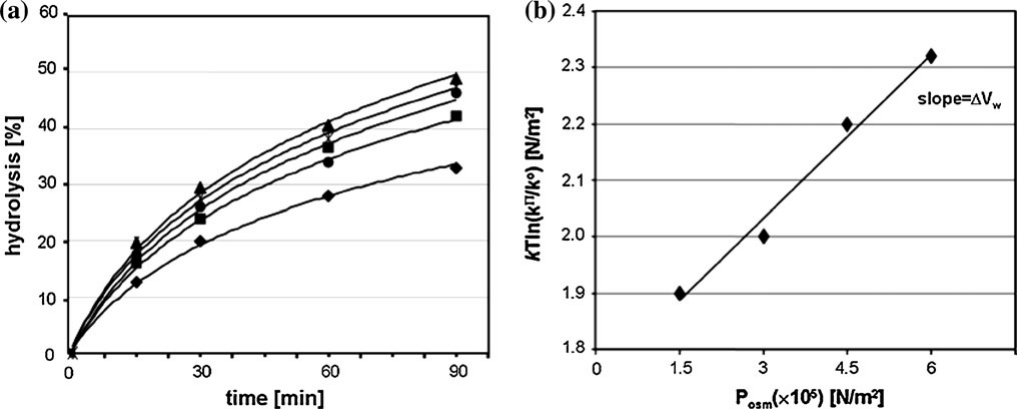
OP dependence of spontaneous hydrolysis of L1. **a** Kinetics of cleavage reaction was carried out in increasing concentration of PEG 400: 0% (*diamonds*), 2.5% (v/v) (*squares*), 5% (*circles*), 7.5% (*crosses*) and 10% (*triangles*). **b** Determination of ΔV_w_ for spontaneous hydrolysis

In the presence of increasing concentration of PEG400 (0–7.5%) the rate of leadzyme catalyzed cleavage reaction increases (Fig. 7a). Based on the plot of ln[k^π/^k^o^] versus OP (Fig. 7b) it was calculated that the formation of the transition state involves the release of 116 water molecules per RNA molecule. However in addition to the specific lead-dependent cut at the C–G bond of the target RNA an additional cleavage between C–A in the double stranded fragment was observed. This cleavage is Pb(II) independent and occurs due to spontaneous hydrolysis of RNA (Fig. 5a, b) [27]. The cleavage of bond between C–A in RNA depends on conformational context [36]. To check the hydration changes accompanying that spontaneous hydrolysis we analyzed the effect of HHP on L1 hydrolysis carried out in the absence of Pb(II). The ΔV^≠^ and ΔV for that cleavage is 10.5 and 2.5 cm^3^/mol, respectively (Fig. 8; Table 1). Determination of ΔV_w_ allows calculation that non-specific cleavage is accompanied by release of 30 water molecules (Fig. 9a, b; Table 1). It means that Pb(II) induced hydrolysis causes release of 86 water molecules, which is pretty close to the amount obtained for tRNA^Phe^.

For the hairpin ribozyme it has been shown that formation of transition state is accompanied by reduction of solvent accessible area (saa) by 1,570 Å^2^ and release of 78 water molecules (~H_2_O/20 Å^2^) [30]. Single base covers an area of approximately 177 Å^2^ [37] thus saa in the case of hairpin corresponds to 8.8 bases. Simple calculation gives ~11 bases (98H_2_O × 20 Å^2^ = 1,960 Å^2^) for tRNA^Phe^, ~9.7 bases (86H_2_O × 20 Å^2^ = 1,720 Å^2^) for leadzyme and ~3.4 (30H_2_O × 20 Å^2^ = 600 Å^2^) in the case of non-specific hydrolysis in helical part of leadzyme. It suggests that water release observed as a result of P–O cleavage was located around the reaction site rather than scattered throughout the molecule. In the case of structurally more simple molecule, like leadzyme, that nine bases form loop and two flanking bp. In more complicated structure like tRNA^Phe^ saa comprises 11 bases, but one have to keep in mind that reaction center consists of two interacting loops TΨC binding Pb^2+^ and DHU (target). However that fragment of tRNA^Phe^ molecule is more hydrated than the remaining part as shown in crystal structure [6].

ΔV^≠^ value for leadzyme catalyzed reaction is close to that obtained for tRNA^Phe^, but ΔV = 24.9 cm^3^/mol for leadzyme is almost two times greater than observed for tRNA^Phe^. It may explain almost a complete inhibition of leadzyme activity at 400 MPa, while for tRNA^Phe^ ca. 2.5% of hydrolysis was still detected at 800 MPa after 2 h (Figs. 2a, 6a).

The non-linear plot of lnk_obs_ versus pressure for tRNA^Phe^ means that the activation isothermal compressibility factor (Δβ^≠^) cannot be neglected (Fig. 2c) [32]. Considering the differences between that plot for tRNA^Phe^ and leadzyme (Figs. 2b, 6b, 8b) one has to keep in mind difference in the size of both molecules as well as in mechanism of the reaction. The plot of lnk_obs_ versus pressure depends on: (i) pressure-induced quaternary structure change (dissociation of subunits, changes of the architecture of binding and active sites), (ii) changes in compressibility (including changes in hydration) and (iii) changes in the rate-determining step [32]. The pressure induced unfolding and dissociation of subunits which are generally observed for proteins does not occur in the case of nucleic acids [38, 39]. So positive value of isothermal compressibility changes (Δβ_app_^≠^ 0.064 cm^3^/mol) is associated with compression of cavities in the macromolecule interior, changes in intramolecular packing and increase in molecule hydration [32]. An increase of yeast tRNA^Phe^ hydration and changes of its structure were observed with FTIR [33]. However previous considerations does not exclude the influence of pressure on rate-determining step. The first step of Pb(II) induced hydrolysis of RNA is the same in both cases tRNA^Phe^ and leadzyme [15, 20]. Spontaneous hydrolysis observed in helical fragment of RNA proceeds according to the same mechanism [40]. While in the case of small metalloribozyme the reaction proceeds according to two-step mechanism and in the second step the 2′,3′-cyclic phosphodiester is further converted to 3′-monophosphate [20]. So the difference observed between tRNA^Phe^ and leadzyme might results from the different mechanism of reaction.

Our results show that cleavage of RNA with Pb(II) is pressure inhibited while spontaneous cut of RNA occurring in helical region is relatively less HHP dependent. It means that structural changes of RNA during in Pb(II) induced hydrolysis are bigger. Hydrolysis of single phosphodiester bond in the loop of RNA is accompanied by three times higher hydration changes comparing to cleavage in helix. The first step of volume change of tRNA^Phe^ caused by increasing pressure is connected with decrease of water volume while further effects arise from tRNA conformational changes.

## Abbreviations

HHP: High hydrostatic pressure
OP: Osmotic pressure
AP: Ambient pressure
